# Efficacy of levofloxacin against biofilms of *Pseudomonas aeruginosa* isolated from patients with respiratory tract infections in vitro

**DOI:** 10.1002/mbo3.720

**Published:** 2018-09-05

**Authors:** Pengfei She, Zhen Luo, Lihua Chen, Yong Wu

**Affiliations:** ^1^ Department of Clinical Laboratory The Third Xiangya Hospital of Central South University Changsha China

**Keywords:** biofilm, levofloxacin, *Pseudomonas aeruginosa*, respiratory tract infection, RNA sequencing

## Abstract

Microbial biofilms are formed in a variety of clinical situations and increase antibiotic resistance of the pathogen by almost ~1,000 times. The effect of levofloxacin (OFLX) on the biofilms of *Pseudomonas aeruginosa* strain PAO1 and the clinical isolates was investigated by crystal violet staining and confocal laser scanning microscope. The transcriptional alteration in the PAO1 biofilms upon OFLX treatment was also analyzed by RNA sequencing (RNA‐*seq*). We found that while OFLX significantly inhibited *P. aeruginosa* biofilm formation in a dose‐dependent manner, it could not completely eradicate preformed biofilms even at higher concentrations. RNA‐*seq* revealed that PAO1 genes related to metabolism, formation of secondary metabolites, and quorum sensing biosynthesis were differentially expressed in the biofilms treated with OFLX. Our data might be useful in determining the optimum OFLX concentration needed for *P. aeruginosa* biofilm inhibition and eradication in patients with respiratory tract infections.

## 1 INTRODUCTION

Biofilm is a three‐dimensional structured community of microbial cells that adhere to a biotic or abiotic surface and are enclosed in a self‐produced polymeric matrix (Costerton, Stewart, & Greenberg, 1999). Biofilm formation is initiated by adherence of single planktonic cells to a surface, leading to the development microcolonies, which then further grow into mature biofilms that produce components of the extracellular matrix (ECM) to maintain the distinctive structures of cellular aggregates (Hentzer et al., 2003). They act as barriers to antimicrobial agents and protect the colonies from any environmental fluctuations. When encased in a biofilm, bacteria can be almost 1,000‐fold more resistant to antibiotics compared with their planktonic counterparts, often rendering the antibiotic therapy ineffective (Chu et al., 2016; Drilling et al., 2014). This is probably due to the slow growth, metabolic shift, production of ECM, and different cell surface properties of the embedded bacteria (Stryjewski & Corey, 2014). According to published reports, over 80% microbial infections in humans are due to biofilms (Musk & Hergenrother, 2006).

With the increasing number of immunocompromised patients, the clinical relevance of opportunistic pathogens has increased in recent years. *Pseudomonas aeruginosa* is one of the most important opportunistic human pathogens (Sousa & Pereira, 2014). These gram‐negative bacteria kill thousands of people annually and are responsible for 10% of all hospital‐acquired infections (Davies et al., 1998). The biofilms of *P. aeruginosa* play a substantial role in cystic fibrosis pneumonia, chronic wound infections, chronic otitis media, chronic bacterial prostatitis, and medical device‐related infections (Rybtke, Hultqvist, Givskov, & Tolker‐Nielsen, 2015).

Levofloxacin (OFLX) belongs to a new class of fluoroquinolones, and its activity on *Stretococcus pneumoniae* and other respiratory pathogens has been widely studied and documented (Marchetti & Viale, 2003). However, the inhibitory action of OFLX against *P. aeruginosa* biofilms is still controversial due to the different drug concentrations, duration of treatment, and the age of biofilms in different studies. To the best of our knowledge, there is no report so far that evaluates the effect of OFLX on biofilm transcriptomics. We have examined the inhibitory effects of OFLX on the biofilms of *P. aeruginosa* strain PAO1 and clinical isolates by crystal violet (CV) staining and confocal laser scanning microscopy (CLSM), along with the morphological changes in the biofilms associated with OFLX therapy. To determine the potential mechanisms of the antibiofilm effect of OFLX, we analyzed the differentially expressed genes in the biofilms using high‐throughput RNA‐*seq*.

## 2 MATERIALS AND METHODS

### 2.1 Bacterial strains, growth media, and conditions

Type strain PAO1 (ATCC 15692) used in this study was kindly provided by Mingqiang Qiao (College of Life Sciences of Nankai University, Tianjin, China). And *P. aeruginosa* ATCC 27853 was preserved by our laboratory. The clinical isolates of *P. aeruginosa* were isolated from the patients with pulmonary infections at the third Xiangya Hospital of Central South University (Changsha, Hunan, China) during January 2014 to December 2014 (Qu et al., 2016). All of the strains were stored at −80°C in whole milk culture. And Luria‐Bertani (LB) broth (Solarbio, Shanghai, China) was used for bacterial culture in all experiments.

### 2.2 Materials

OFLX (Aladdin, Shanghai, China) was used and dissolves in distilled water according to the Clinical and Laboratory Standards Institute (CLSI) criteria (2015) (Clinical and Laboratory Standards Institute, 2015). The final concentration of OFLX stock solution was 6.4 mg/ml.

### 2.3 Determination of the minimal inhibitory concentration

The minimal inhibitory concentration (MIC) was detected by the broth microdilution method as previously described by The CLSI 2015. Briefly, twofold dilutions of OFLX (ranging from 1,024 to 0.0625 μg/ml) were prepared in Mueller‐Hinton broth, and 100 μl was dispensed per well in a 96‐well plate. Each well was then inoculated with 10 μl of *P. aeruginosa* suspension, and the plates were incubated at 35°C for 16~18 hr. The MIC was determined as the lowest concentration without any visible bacterial growth. McFarland standard 0.5 was used as the reference for turbidity.

### 2.4 Biofilm‐forming capacity

*Pseudomonas aeruginosa* biofilms were grown according to a previously published protocol (Nesse, Berg, & Vestby, 2015). Briefly, an overnight culture of *P. aeruginosa* was diluted 1:200 with LB broth, and 200 μl aliquots were dispensed per well in microplates (Corning/Costar, USA). After 16‐hr incubation at 37°C without shaking, the plates were washed gently with 0.9% saline to remove planktonic cells and then stained with 200 μl 0.25% (w/v) CV solution. After incubation at room temperature for 15 min, the unbound CV was removed with saline, and 200 μl 95% ethanol was added to dissolve the stained dye. Absorbance was measured at 570 nm, and biofilm‐forming capacity was calculated as *A*_570 nm_. The biofilm‐forming ability was classified according to the criteria of Hassan et al. (2011) (Table 1).

**Table 1:** Classification of biofilm production

Average *A*_570 nm_ value	Biofilm production
≤*A*_c_	Non
*A*_c_ < ~ ≤ 2 × *A*_c_	Weak
2 × *A*_c_ < ~ ≤ 4 × *A*_c_	Moderate
>4 × *A*_c_	Strong

*Note*

Absorbance cutoff value (*A*_c_) = average *A*_570 nm_ of negative control (ATCC27853) + 3 × standard deviation (*SD*) of negative control.

### 2.5 Biofilm inhibition and eradication assay

The biofilm inhibitory assay was performed in microplates as previously described (Qu et al., 2016). Briefly, 4 μl overnight culture and 196 μl LB broth were dispensed per well in a 96‐well microplate, and exposed to different concentrations of OFLX. After incubation for 24 hr at 37°C, biofilm biomass was determined by the CV staining method as described. For biofilm eradication (Christensen et al., 1985), overnight culture was diluted 1:200 with LB broth, and 100 μl bacterial suspension was dispensed per well in microplates. After static incubation for 24 hr at 37°C, the planktonic cells were removed with saline, and 200 μl LB broth with OFLX was added to each well. After incubation for another 24 hr, biofilm biomass was detected with CV staining.

### 2.6 CLSM observation

In order to investigate the biofilm characteristics affected by OFLX, glass slides were placed in six‐well microplates containing 2 ml LB broth with OFLX, and 40 μl overnight culture was added per well. After incubation for 24 hr, the planktonic cells were removed and the biofilms remaining on the glass slides were stained with LIVE/DEAD BacLight Bacterial Viability Kit (Thermo Fisher Scientific, Shanghai, China) as recommended. Briefly, 1.5 μl of SYTO green and PI red dye mix was diluted in 1 ml PBS (pH 7.4), and 100 μl of the dye solution was dropped on each glass slide. After incubation in the dark for 15 min, the glass slides were observed by CLSM (Zeiss LSM 800, Jena, Germany), and the biofilm biomass was quantified with ImageJ software.

### 2.7 RNA sequencing

*Pseudomonas aeruginosa* PAO1 was incubated in LB broth with or without 1 μg/ml OFLX (MIC = 2 μg/ml) for 16 hr. After removing the planktonic cells, the biofilms were collected with moist swabs. Three independent biological replicates were used for further analysis. The total RNA was extracted from the biofilms with E.Z.N.A Total RNA Kit II. Subsequent transcriptomic analysis was carried out by the BGI Company. Briefly, sequencing reads’ filtering was performed by the SOAPnuke program, and clean reads were mapped to the reference genome of *P. aeruginosa* (RefSeq NC_002516.2) using HISAT2 program. The StringTie program (version v1.0.4) was then used to the reconstruct the transcripts, and Cuffcompare was used to compare the transcripts and reference annotation. CPC was used to predict the coding potential of the novel transcripts, and differentially expressed genes (DEGs) were determined by DEGseq, DEseq2, PossionDis, NOIseq, and EBseq analysis. Finally, gene ontology (GO) and Kyoto Encyclopedia of Genes and Genomes (KEGG) pathway classification analysis were performed to determine the function of the DEGs (Slany, Oppelt, & Cincarova, 2017).

### 2.8 Statistical analysis

All experiments were repeated three times to validate the reproducibility. And the data were analyzed by using GraphPad Prism 7.0 software (GraphPad Software, San Diego, CA, USA), and Student's *t* test was used to determine the statistical significance of the data. The level of statistical significance was set at *p *<* *0.05.

## 3 RESULTS

### 3.1 Effects of OFLX on biofilm formation and eradication

**Figure 1:**
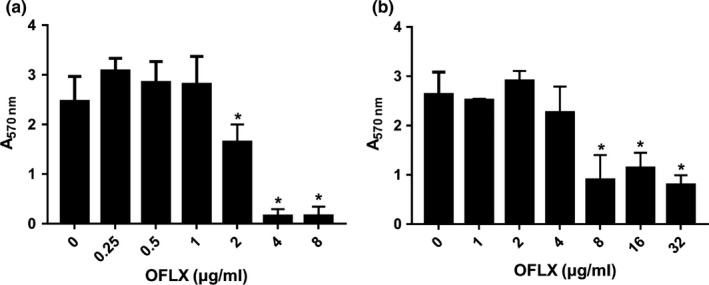
Antibiofilm activity of OFLX on PAO1. The effects of OFLX on biofilm formation (*n* = 3) (a) and eradication (*n* = 3) (b) at different concentrations, respectively. The data are reported as absorbance at 570 nm (*A*_570 nm_) of residual biofilm

The effects of OFLX on PAO1 biofilm formation and its eradication are shown in Figure 1. Treatment with OFLX at the MIC (2 μg/ml) significantly inhibited PAO1 biofilm formation and continued its inhibitory effect in a dose‐dependent manner (Figure 1a). However, higher concentrations of OFLX were needed to eradicate the preformed biofilms. Although biofilms were significantly eradicated by OFLX at 8 μg/ml, there was no significant decrease in the biofilm biomass even at the concentration of 32 μg/ml (Figure 1b).

### 3.2 Biofilm‐forming capacity of pulmonary isolates

**Figure 2:**
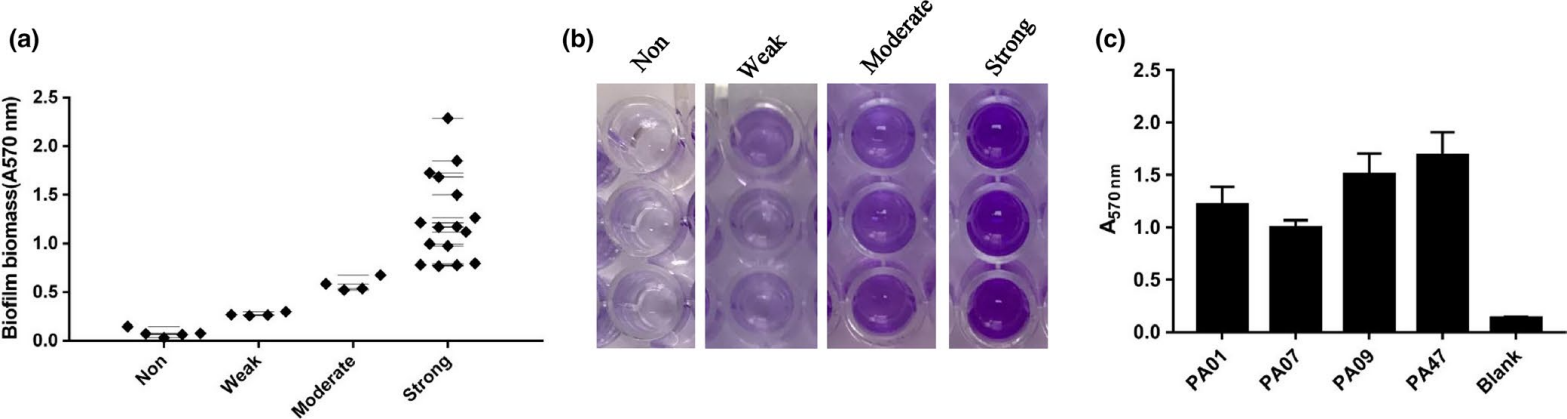
Biofilm‐forming capacity of *Pseudomonas aeruginosa* pulmonary isolates. Biofilm was formed in microplates at 37°C for 24 hr and stained with crystal violet. (a) Distribution of biofilm production in clinical isolates (*n* = 29). (b) Representative images of biofilm formation by CV staining. (c) Biofilm‐forming ability of strains chosen for our following study. The data are reported as *A*_570nm_ of residual biofilm

OFLX tolerance of the biofilms of 30 clinical isolates of *P. aeruginosa*, isolated from the sputum or respiratory tract irrigate of pulmonary infection patients, was tested. CV staining identified five (16.67%) strains as biofilm negative, four (13.33%) as weak, four (13.33%) as moderate, and 17 (56.67%) as strong biofilm producers (Figure 2a), which was consistent with the report of Olson et al. (2002). In addition, significant differences were seen among different biofilm‐forming groups by CV staining (Figure 2b). Of the 17 strong biofilm producers, four strains (PA01, PA07, PA09, and PA47) were selected for further experiments (Figure 2c).

### 3.3 Antibiofilm effect of OFLX on pulmonary isolates

**Figure 3:**
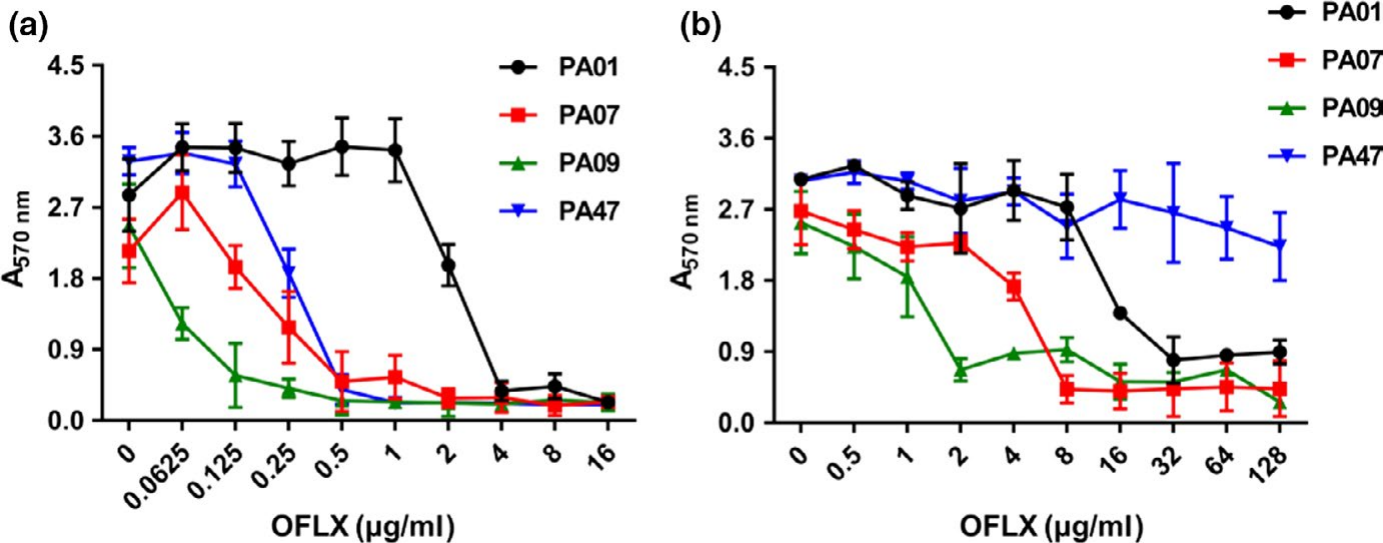
Antibiofilm effect of OFLX on strong biofilm producers of *P. aeruginosa* clinical isolates. The effects of OFLX at different concentrations on biofilm formation (a) and eradication (b) of *P. aeruginosa* isolated from patients with pulmonary infections. The data are reported as *A*_570nm_ of residual biofilm

Biofilms of different clinical isolates showed different susceptibility patterns to OFLX. It significantly inhibited the biofilm formation of PA01, PA07, PA09, and PA47 strains at the respective MIC values (2, 0.25, 0.0625, and 0.25 μg/ml for PA01, PA07, PA09, and PA47, respectively; *p *<* *0.05), as well as in a dose‐dependent manner (Figure 3a). PA47 was the strongest biofilm producer among them, but the biofilm of PA01 showed the highest resistance to OFLX. However, biofilms of all strains showed higher resistance to complete eradication by OFLX. It could significantly eradicate the biofilms of PA01, PA07, PA09, and PA47 at the concentrations of 16, 4, and 2 μg/ml, respectively (Figure 3b). In addition, the 24‐hr biofilm of PA47 was fully resistant to OFLX even at 128 μg/ml. Finally, there was no significant decrease in the preformed biofilm biomass with increasing concentration of OFLX.

### 3.4 Morphological characteristics of *P. aeruginosa* biofilms

**Figure 4:**
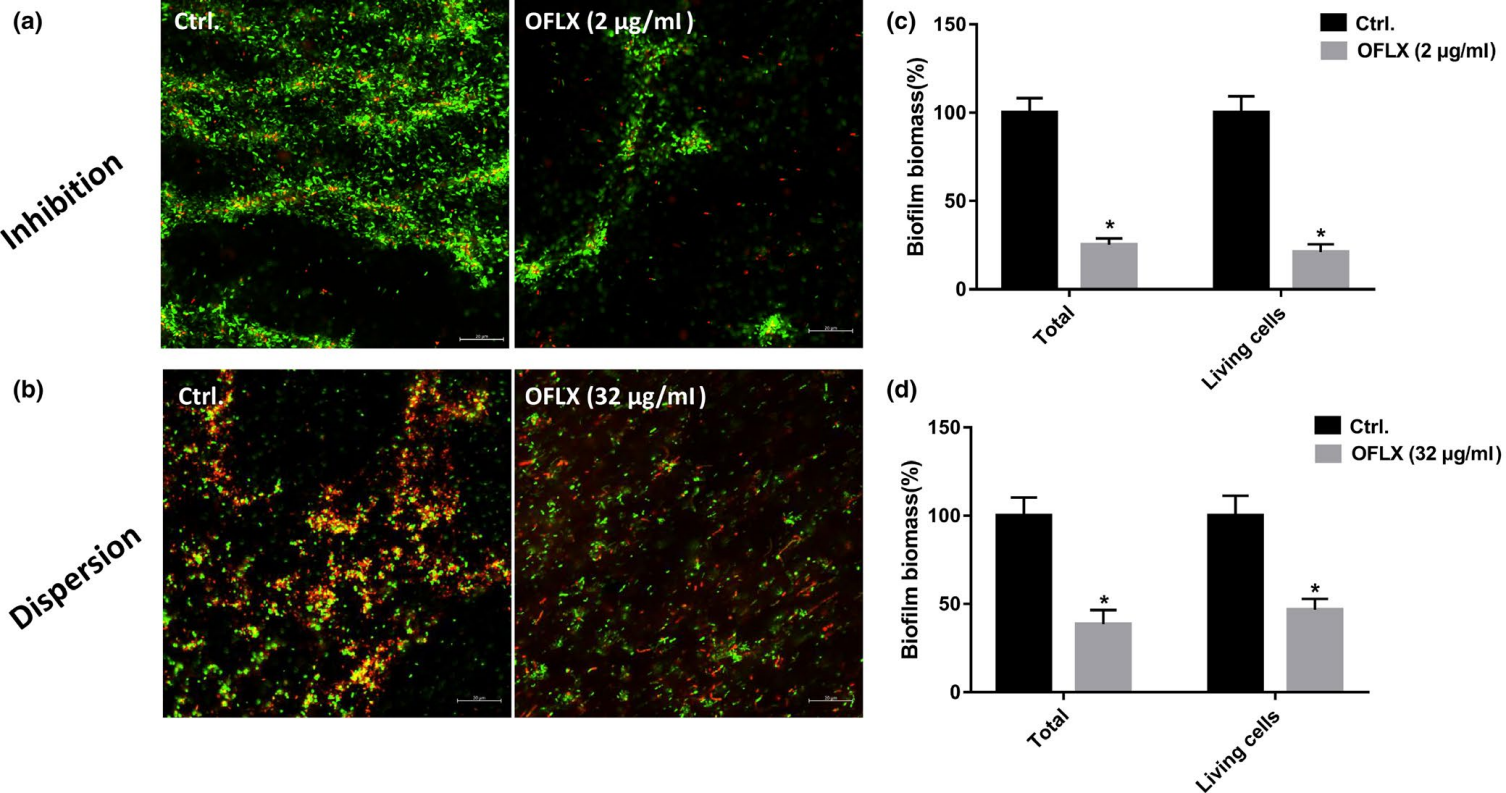
CLSM of PAO1 biofilms in the presence of OFLX. Biofilm inhibition (a) and eradication (b) by OFLX at the concentration of 2 and 32 μg/ml, respectively. Then, quantification of total and living biofilm biomass on cover slides in biofilm inhibition assay (c) and eradication assay (d) was performed by ImageJ software. PAO1 grown on cover slides was stained with LIVE/DEAD reagents, and the green (SYTO9) and red (PI) fluorescence indicate viable and dead cells, respectively

**Figure 5:**
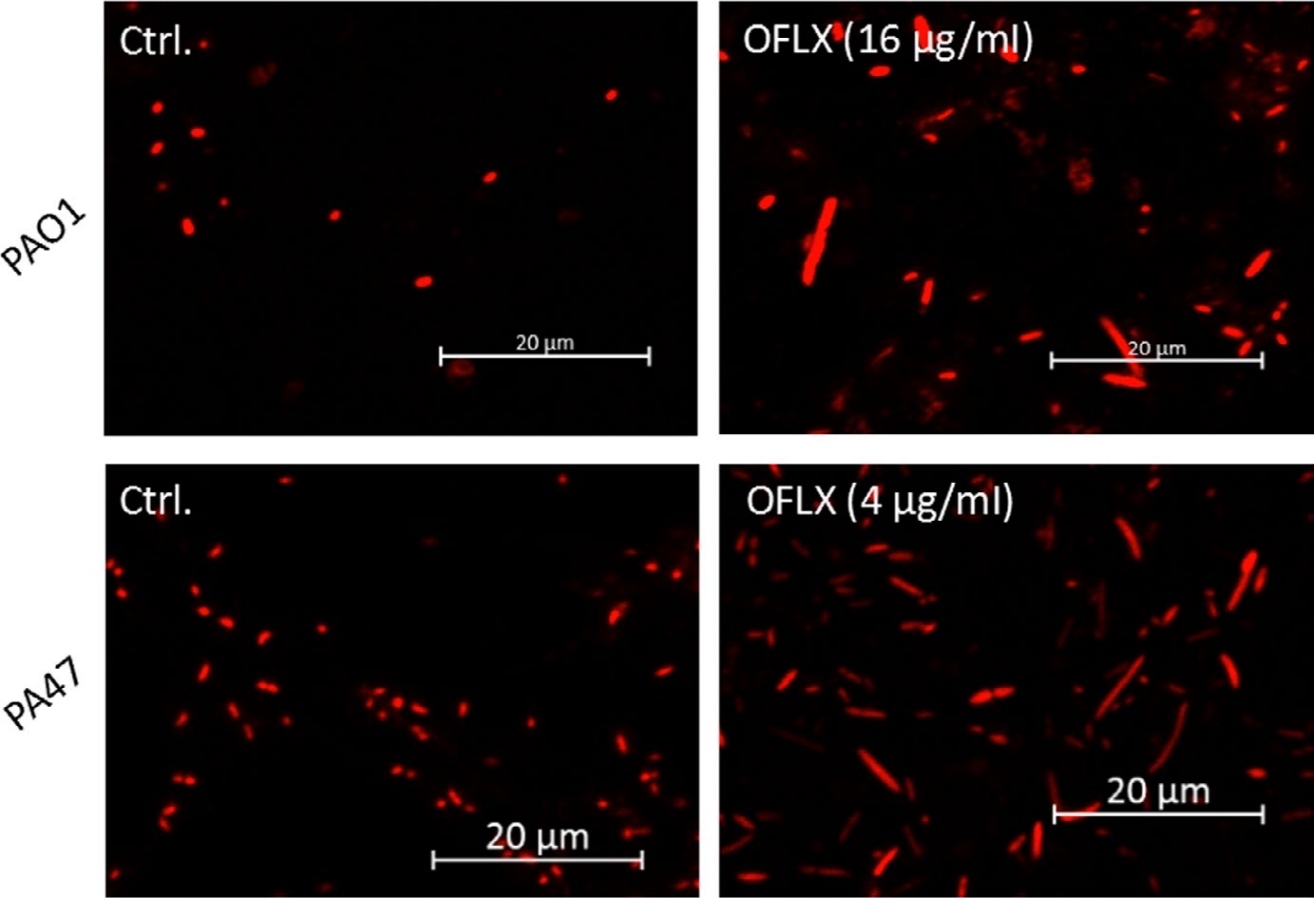
*Pseudomonas aeruginosa* morphological alteration by OFLX treatment. OFLX elongated the cells of PAO1 and PA47 at the concentration of 16 and 4 μg/ml, respectively. PAO1 grown on cover slides in the presence or absence of OFLX were stained with red fluorescence (PI)

While the untreated biofilms were thick and relatively homogeneous, treatment with 2 μg/ml OFLX significantly decreased the biofilm biomass and disrupted its structural integrity (Figure 4a,c). OFLX could slightly disrupt the integrity of preformed biofilms (Figure 4b) and even eradicated the bulk of the biomass to some extent, but living cells remained even at the concentration of 32 μg/ml (Figure 4d). In addition, significant morphological alterations were observed in the PAO1 strain and the clinical isolate PA47 following OFLX treatment (Figure 5). Rounded or rod‐shaped cells were observed in the untreated sample, while elongated cells with or without swelling appeared in the presence of OFLX.

### 3.5 OFLX‐inhibited biofilms exhibit a novel transcriptome

**Figure 6:**
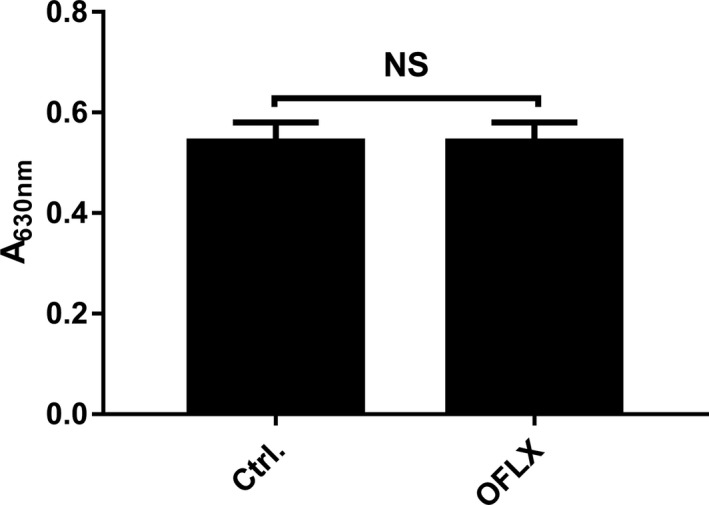
Effects of OFLX on planktonic cells growth of PAO1 at the concentration of 1 μg/ml. NS: no statistically significance

**Figure 7:**
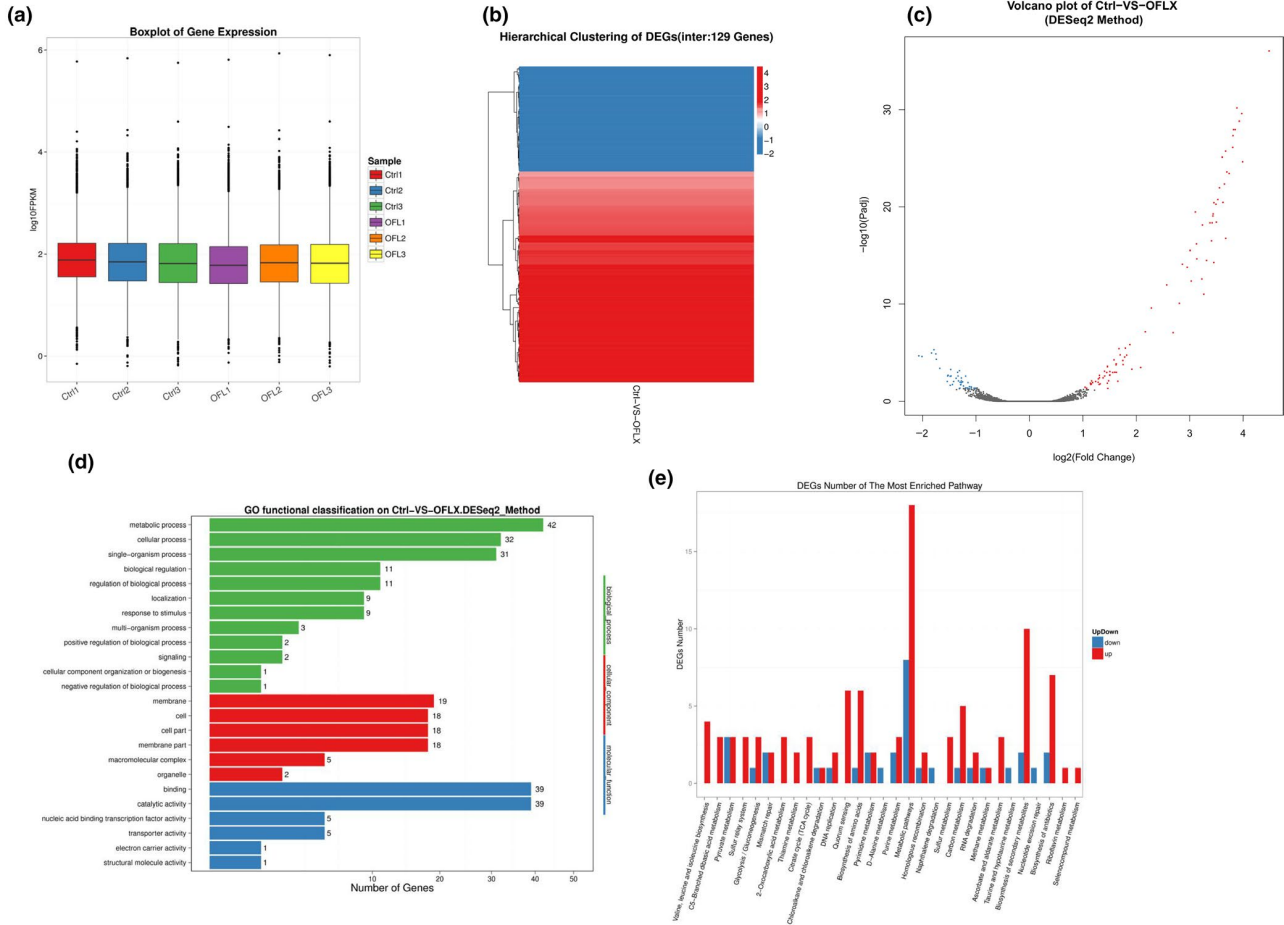
RNA sequencing analysis. (a) The box plot shows the variations in mRNA expression. (b) Heatmap showing gene expression patterns. (c) Volcano plots of DE genes for PAO1 biofilm formed in response to 1 μg/ml OFLX. And the red, blue, and gray dots indicate upregulated, downregulated, and nonregulated genes, respectively. GO analysis (d) and KEGG pathway classification analysis (e) of upregulated and downregulated genes

In order to avoid the effect of cell growth inhibition on the expression of mRNA, OFLX at sub‐MIC (1 μg/ml) was chosen for RNA‐*seq* analysis (Figure 6). Sequencing analysis revealed significant transcriptional changes in the PAO1 biofilms after a 16‐hr treatment with OFLX (Figure 7). The raw expression levels of each sample are shown in the boxplots, and little variability was observed between the samples (Figure 7a). A total of 129 differentially expressed genes (DEGs) were seen in the OFLX‐treated biofilms, compared to the untreated control group (Figure 7b), of which 86 were upregulated and 43 downregulated (Figure 7c). GO function classification analysis was performed to identify alterations in the biological processes (BP), cellular composition (CC), and molecular functions (MF), which revealed that the DEGs were mainly related to metabolic processes (42 DEGs), cellular processes (32 DEGs), single‐organism processes (31 DEGs), molecular binding (39 DEGs), and catalytic activity (39 DEGs) (Figure 7d). Furthermore, KEGG pathway classification analysis showed that the DEGs were mainly enriched in the pathways of metabolism, secondary metabolites biosynthesis, quorum sensing, and amino acids biosynthesis (Figure 7e).

## 4 DISCUSSION

*Pseudomonas aeruginosa* can adhere to the surface of the respiratory tract and form a biofilm, which is considered to be major cause of refractory pulmonary infections such as cystic fibrosis and panbronchiolitis which are very difficult to cure (Cai et al., 2016). The bacteria in the biofilms are enclosed in a hydrated matrix of extracellular polymeric substances composed of polysaccharides, nucleic acids, lipids, proteins, etc. (Hoiby, 2017). Bacteria within a biofilm are more resistant to antimicrobial agents compared with the planktonic forms (Prosser, Taylor, Dix, & Cleeland, 1987), resulting in excess morbidity and mortality as well as higher healthcare costs (Noreddin & Elkhatib, 2009). The prevalence of biofilms in pulmonary infection is 83.33%, as a high proportion of *P. aeruginosa* strains are strong biofilm producers.

Fluoroquinolones have been used traditionally against the slow‐growing and nongrowing bacteria (Eng, Padberg, Smith, Tan, & Cherubin, 1991), and OFLX has shown a considerable killing effect on *P. aeruginosa* biofilms in experimental studies (Mikuniya et al., 2005). In the present study, OFLX significantly inhibited biofilm formation of both PAO1 and respiratory isolates of *P. aeruginosa*, but showed moderate or slight inhibitory effects on the preformed biofilms. In addition, in accordance with the previous studies (Monden, Ando, Iida, & Kumon, 2002; Nielsen & Nielsen, 1987), the round PAO1 and PA47 cells were elongated in the presence of OFLX. Finally, RNA‐*seq* indicated differential gene expression in PAO1 biofilms upon OFLX treatment.

OFLX interfered with *P. aeruginosa* biofilm synthesis in a dose‐dependent manner. In agreement with our results, Fernández‐Olmos et al. (2012) reported that fluoroquinolones, in particular OFLX, had the most potent effect against the biofilms of *P. aeruginosa* isolates of chronic obstructive pulmonary disease. The findings of Abdi‐Ali, Mohammadi‐Mehr, and Agha Alaei (2006) also support our conclusions. The possible mechanisms of action of OFLX on *P. aeruginosa* biofilms include inhibition of new glycocalyx formation, release, or activation of exopolysaccharide decomposition enzymes, and electrostatic interference with bacterial adhesion (Eng et al., 1991). Therefore, OFLX activity on early attached cells might prevent further biofilm development.

However, OFLX had limited inhibitory effect on the preformed and well‐established biofilms. This observation is also consistent with Fernández‐Olmos et al. (2012) who found that higher concentrations of antibiotics were needed to eradicate the preformed biofilms. Our results reaffirm the higher resistance of OFLX in performed biofilms of *P. aeruginosa*. However, it is not easy to maintain such high concentrations of an antibiotic for a considerable length of time in various clinical situations. In addition, the accumulation kinetics of OFLX in biofilm cells is likely more complex compared to that in planktonic cells. The possible mechanisms of the higher resistance of biofilms to OFLX treatment are (a) blocked access of the antibiotic into the embedded bacterial cells by the ECM, (b) partial or complete replacement of the drug‐susceptible population with resistant mutants over time, (c) emergence of persistent cells (Mulcahy, Burns, Lory, & Lewis, 2010), and (d) formation of small colony variants (Evans, 2015), as well as other unknown resistance mechanisms. Once the antibiotic is removed, the remaining biofilm resumes growth and returns to its initial size (Drago et al., 2011).

CLSM showed significant elongation of *P. aeruginosa* cells in the biofilms following OFLX treatment. In a study by Monden et al. (2002), scanning electron microscope, transmission electron microscope, and CLSM were used to investigate the morphological changes in *P. aeruginosa* biofilms in the presence of OFLX and fosfomycin. They also demonstrated a clear OFLX‐induced elongation of the surface biofilm cells, but this change was not clear in the embedded cells. Since fluoroquinolones disrupt the membrane potential or electron transport (Celesk & Robillard, 1989), it is possible that changes in the bacterial outer membrane lead to changes in cell wall synthesis and fluidity (Kahan, Kahan, Cassidy, & Kropp, 1974). However, little is known about the specific mechanisms and consequences of the shape change in these cells.

*Pseudomonas aeruginosa* biofilms showed significant changes in their transcriptomic profiles after OFLX treatment compared to the untreated cells, with a total of 129 DEGs. Contrary to our expectations, there were only a few of these DEGs that were associated with the well‐known biofilm formation‐related genes like the quorum sensing genes (*lasI/R*,* rlhI/R*,* pqsR et*c.) (Lee & Zhang, 2015) and cyclic diguanylate cyclase expression‐associated genes (*siaA* and *siaD*) (Chen et al., 2015). Instead, most of the DEGs did not have any specific function. It is possible that instead of a unique influential factor, the inhibitory effects of OFLX on *P. aeruginosa* biofilm synthesis depend on multiple factors. KEGG pathway analysis revealed that the DEGs mainly contributed to metabolic processes, secondary metabolites, and amino acid biosynthesis.

Interestingly, though OFLX showed significant inhibitory effects on biofilm formation, there was more or equal amount of upregulated genes than downregulated genes among these significantly changed pathways. It is possible that the working concentration of OFLX we used was 1 μg/ml. And the sub‐MIC of OFLX used in the transcriptomic analysis resulted in stronger expression of antibiotic resistance‐related genes rather than inhibition of biofilm synthesis‐related genes. Cipriani, Giordano, Magni, Papa, and Filadoro (1995) reported that *P. aeruginosa* showed significant changes in their outer membrane and a 32‐fold increase in MIC values, after a five‐day exposure to sub‐MIC of ciprofloxacin. Similarly, Shun‐Mei et al. (2018) found that after treating *E. coli* with sub‐MIC of OFLX, the antibiotic resistant gene transfer frequency was significantly enhanced by upregulation of conjugation‐associated genes via an SOS‐independent mechanism. In addition, Bhattacharya, Dey, Das, and Banerjee (2017) showed that sub‐MIC levels of antibiotics can lead to resistance and cross‐resistance across several classes of antibiotics in wild strains of *S. aureus*, possibly by free radical production. Finally, Gupta, Chhibber, and Harjai (2016) found that sub‐MIC of ciprofloxacin could inhibit *P. aeruginosa* biofilm formation and virulence factor production by targeting the QS system. However, in our study, the QS‐related pathway was significantly upregulated in the presence of sub‐MIC OFLX, indicating different mechanisms of inhibiting *P. aeruginosa* biofilm formation by ciprofloxacin and OFLX at sub‐MIC, though both are fluoroquinolones.

In summary, OFLX can inhibit *P. aeruginosa* biofilm synthesis in a dose‐dependent manner, but cannot fully eradicate the preformed biofilms even at high concentrations. In addition, 1 μg/ml OFLX significantly altered the expression of PAO1 genes related to metabolic processes, cellular processes, single‐organism processes, molecular binding, and catalytic activity. Our data can help determine the optimum OFLX concentration needed for biofilm inhibition and eradication of *P. aeruginosa* infection.

## CONFLICT OF INTEREST

None declared.

## AUTHORS CONTRIBUTIONS

She P and Wu Y designed experiments. She P and Luo Z carried out experiments. Chen L analyzed experimental results. She P and Wu Y wrote the manuscript.

## Data Availability

The raw data of transcriptome sequencing for control group and OFLX‐treated group were shown in Supporting Information Tables [Link], [Link], [Link] and Supporting Information Tables [Link], [Link], [Link], respectively. And the gene different expression analysis was shown in Supporting Information Table S7.

## References

[mbo3720-bib-0001] Abdi‐Ali, A. , Mohammadi‐Mehr, M. , & Agha Alaei, Y. (2006). Bactericidal activity of various antibiotics against biofilm‐producing *Pseudomonas aeruginosa* . International Journal of Antimicrobial Agents, 27, 196–200. 10.1016/j.ijantimicag.2005.10.007 16459057

[mbo3720-bib-0002] Bhattacharya, G. , Dey, D. , Das, S. , & Banerjee, A. (2017). Exposure to sub‐inhibitory concentrations of gentamicin, ciprofloxacin and cefotaxime induces multidrug resistance and reactive oxygen species generation in meticillin‐sensitive *Staphylococcus aureus* . Journal of Medical Microbiology, 66, 762–769. 28598307 10.1099/jmm.0.000492

[mbo3720-bib-0003] Cai, S. , Li, Y. , Wang, K. , Cen, Y. , Lu, H. , Dong, B. , … Kong, J. (2016). Pathogenic effects of biofilm on *Pseudomonas Aeruginosa* pulmonary infection and its relationship to cytokines. Medical Science Monitor, 22, 4869–4874. 10.12659/MSM.898783 27941713 PMC5156558

[mbo3720-bib-0004] Celesk, R. A. , & Robillard, N. J. (1989). Factors influencing the accumulation of ciprofloxacin in *Pseudomonas aeruginosa* . Antimicrobial Agents and Chemotherapy, 33, 1921–1926. 10.1128/AAC.33.11.1921 2514623 PMC172788

[mbo3720-bib-0005] Chen, Y. , Yuan, M. , Mohanty, A. , Yam, J. K. , Liu, Y. , Chua, S. L. , … Yang, L. (2015). Multiple diguanylate cyclase‐coordinated regulation of pyoverdine synthesis in *Pseudomonas aeruginosa* . Environmental Microbiology Reports, 7, 498–507. 10.1111/1758-2229.12278 25683454

[mbo3720-bib-0006] Christensen, G. D. , Simpson, W. A. , Younger, J. J. , Baddour, L. M. , Barrett, F. F. , Melton, D. M. , & Beachey, E. H. (1985). Adherence of coagulase‐negative staphylococci to plastic tissue culture plates: A quantitative model for the adherence of staphylococci to medical devices. Journal of Clinical Microbiology, 22, 996–1006. 3905855 10.1128/jcm.22.6.996-1006.1985 PMC271866

[mbo3720-bib-0007] Chu, M. , Zhang, M. B. , Liu, Y. C. , Kang, J. R. , Chu, Z. Y. , Yin, K. L. , … Wang, Y. D. (2016). Role of berberine in the treatment of methicillin‐resistant *Staphylococcus aureus* infections. Scientific Reports, 6, 24748 10.1038/srep24748 27103062 PMC4840435

[mbo3720-bib-0008] Cipriani, P. , Giordano, A. , Magni, A. , Papa, F. , & Filadoro, F. (1995). Outer membrane alterations in *Pseudomonas aeruginosa* after five‐day exposure to quinolones and carbapenems. Drugs Under Experimental and Clinical Research, 21, 139–144. 8529526

[mbo3720-bib-0009] Clinical and Laboratory Standards Institute (2015). Performance standards for antimicrobial susceptibility testing. 23th informational supplement CLSI document M100‐S25, vol. 35, No 3.

[mbo3720-bib-0010] Costerton, J. W. , Stewart, P. S. , & Greenberg, E. P. (1999). Bacterial biofilms: A common cause of persistent infections. Science, 284, 1318–1322. 10.1126/science.284.5418.1318 10334980

[mbo3720-bib-0011] Davies, D. G. , Parsek, M. R. , Pearson, J. P. , Iglewski, B. H. , Costerton, J. W. , & Greenberg, E. P. (1998). The involvement of cell‐to‐cell signals in the development of a bacterial biofilm. Science, 280, 295–298. 10.1126/science.280.5361.295 9535661

[mbo3720-bib-0012] Drago, L. , Mattina, R. , Legnani, D. , Romano, C. L. , Vianello, E. , Ricci, C. , & De Vecchi, E. (2011). Modulation of biofilm of strains isolated from patients with chronic obstructive pulmonary disease by levofloxacin, moxifloxacin, ciprofloxacin, amoxicillin/clavulanic acid and ceftriaxone. International Journal of Immunopathology and Pharmacology, 24, 1027–1035. 10.1177/039463201102400420 22230408

[mbo3720-bib-0013] Drilling, A. , Morales, S. , Boase, S. , Jervis‐Bardy, J. , James, C. , Jardeleza, C. , … Wormald, P. J. (2014). Safety and efficacy of topical bacteriophage and ethylenediaminetetraacetic acid treatment of *Staphylococcus aureus* infection in a sheep model of sinusitis. International Forum of Allergy & Rhinologyis, 4, 176–186. 10.1002/alr.21270 24449635

[mbo3720-bib-0014] Eng, R. H. , Padberg, F. T. , Smith, S. M. , Tan, E. N. , & Cherubin, C. E. (1991). Bactericidal effects of antibiotics on slowly growing and nongrowing bacteria. Antimicrobial Agents and Chemotherapy, 35, 1824–1828. 10.1128/AAC.35.9.1824 1952852 PMC245275

[mbo3720-bib-0015] Evans, T. J. (2015). Small colony variants of *Pseudomonas aeruginosa* in chronic bacterial infection of the lung in cystic fibrosis. Future Microbiology, 10, 231–239. 10.2217/fmb.14.107 25689535

[mbo3720-bib-0017] Fernández‐Olmos, A. , García‐Castillo, M. , Maiz, L. , Lamas, A. , Baquero, F. , & Cantón, R. (2012). *In vitro* prevention of *Pseudomonas aeruginosa* early biofilm formation with antibiotics used in cystic fibrosis patients. International Journal of Antimicrobial Agents, 40, 173–176. 10.1016/j.ijantimicag.2012.04.006 22727530

[mbo3720-bib-0018] Gupta, P. , Chhibber, S. , & Harjai, K. (2016). Subinhibitory concentration of ciprofloxacin targets quorum sensing system of *Pseudomonas aeruginosa* causing inhibition of biofilm formation & reduction of virulence. Indian Journal of Medical Research, 143, 643–651. 27488009 10.4103/0971-5916.187114 PMC4989839

[mbo3720-bib-0019] Hassan, A. , Usman, J. , Kaleem, F. , Omair, M. , Khalid, A. , & Iqbal, M. (2011). Evaluation of different detection methods of biofilm formation in the clinical isolates. Brazilian Journal of Infectious Diseases, 15, 305–311. 10.1016/S1413-8670(11)70197-0 21860999

[mbo3720-bib-0020] Hentzer, M. , Wu, H. , Andersen, J. B. , Riedel, K. , Rasmussen, T. B. , Bagge, N. , … Givskov, M. (2003). Attenuation of *Pseudomonas aeruginosa* virulence by quorum sensing inhibitors. EMBO Journal, 22, 3803–3815. 10.1093/emboj/cdg366 12881415 PMC169039

[mbo3720-bib-0021] Hoiby, N. (2017). A short history of microbial biofilms and biofilm infections. APMIS, 125, 272–275. 10.1111/apm.12686 28407426

[mbo3720-bib-0022] Kahan, F. M. , Kahan, J. S. , Cassidy, P. J. , & Kropp, H. (1974). The mechanism of action of fosfomycin (phosphonomycin). Annals of the New York Academy of Sciences, 235, 364–386. 10.1111/j.1749-6632.1974.tb43277.x 4605290

[mbo3720-bib-0023] Lee, J. , & Zhang, L. (2015). The hierarchy quorum sensing network in *Pseudomonas aeruginosa* . Protein Cell, 6, 26–41. 10.1007/s13238-014-0100-x 25249263 PMC4286720

[mbo3720-bib-0024] Marchetti, F. , & Viale, P. (2003). Current and future perspectives for levofloxacin in severe *Pseudomonas aeruginosa* infections. Journal of Chemotherapy, 15, 315–322. 12962358 10.1179/joc.2003.15.4.315

[mbo3720-bib-0025] Mikuniya, T. , Kato, Y. , Kariyama, R. , Monden, K. , Hikida, M. , & Kumon, H. (2005). Synergistic effect of fosfomycin and fluoroquinolones against *Pseudomonas aeruginosa* growing in a biofilm. Acta Medica Okayama, 59, 209–216. 16286954 10.18926/AMO/31977

[mbo3720-bib-0026] Monden, K. , Ando, E. , Iida, M. , & Kumon, H. (2002). Role of fosfomycin in a synergistic combination with ofloxacin against *Pseudomonas aeruginosa* growing in a biofilm. Journal of Infection and Chemotherapy, 8, 218–226. 10.1007/s10156-002-0186-6 12373484

[mbo3720-bib-0027] Mulcahy, L. R. , Burns, J. L. , Lory, S. , & Lewis, K. (2010). Emergence of *Pseudomonas aeruginosa* strains producing high levels of persister cells in patients with cystic fibrosis. Journal of Bacteriology, 192, 6191–6199. 10.1128/JB.01651-09 20935098 PMC2981199

[mbo3720-bib-0028] Musk, D. J. Jr , & Hergenrother, P. J. (2006). Chemical countermeasures for the control of bacterial biofilms: Effective compounds and promising targets. Current Medicinal Chemistry, 13, 2163–2177. 10.2174/092986706777935212 16918346

[mbo3720-bib-0029] Nesse, L. L. , Berg, K. , & Vestby, L. K. (2015). Effects of norspermidine and spermidine on biofilm formation by potentially pathogenic *Escherichia coli* and *Salmonella enterica* wild‐type strains. Applied and Environment Microbiology, 81, 2226–2232. 10.1128/AEM.03518-14 PMC4345383 25595767

[mbo3720-bib-0030] Nielsen, B. , & Nielsen, H. (1987). Bactericidal effect of polymorphonuclear leukocytes on *Pseudomonas aeruginosa* pre‐incubated in ofloxacin. Acta Pathologica, Microbiologica, et Immunologica Scandinavica Section B Microbiology, 95, 227–232. 10.1111/j.1699-0463.1987.tb03117.x 3118637

[mbo3720-bib-0031] Noreddin, A. M. , & Elkhatib, W. F. (2009). Novel *in vitro* pharmacodynamic model simulating ofloxacin pharmacokinetics in the treatment of *Pseudomonas aeruginosa* biofilm‐associated infections. Journal of Infection and Public Health, 2, 120–128. 10.1016/j.jiph.2009.07.004 20701871

[mbo3720-bib-0032] Olson, M. E. , Ceri, H. , Morck, D. W. , Buret, A. G. , & Read, R. R. (2002). Biofilm bacteria: Formation and comparative susceptibility to antibiotics. Canadian Journal of Veterinary Research, 66, 86–92. 11989739 PMC226988

[mbo3720-bib-0033] Prosser, B. L. , Taylor, D. , Dix, B. A. , & Cleeland, R. (1987). Method of evaluating effects of antibiotics on bacterial biofilm. Antimicrobial Agents and Chemotherapy, 31, 1502–1506. 10.1128/AAC.31.10.1502 3435100 PMC174979

[mbo3720-bib-0034] Qu, L. , She, P. , Wang, Y. , Liu, F. , Zhang, D. , Chen, L. , … Yong, Wu (2016). Effects of norspermidine on *Pseudomonas aeruginosa* biofilm formation and eradication. Microbiologyopen, 5, 402–412. 10.1002/mbo3.338 26817804 PMC4905993

[mbo3720-bib-0035] Rybtke, M. , Hultqvist, L. D. , Givskov, M. , & Tolker‐Nielsen, T. (2015). *Pseudomonas aeruginosa* biofilm infections: Community structure, antimicrobial tolerance and immune response. Journal of Molecular Biology, 427, 3628–3645. 10.1016/j.jmb.2015.08.016 26319792

[mbo3720-bib-0036] Shun‐Mei, E. , Zeng, J. M. , Yuan, H. , Lu, Y. , Cai, R. X. , & Chen, C. (2018). Sub‐inhibitory concentrations of fluoroquinolones increase conjugation frequency. Microbial Pathogenesis, 114, 57–62. 10.1016/j.micpath.2017.11.036 29174700

[mbo3720-bib-0037] Slany, M. , Oppelt, J. , & Cincarova, L. (2017). Formation of *Staphylococcus aureus* biofilm in the presence of sublethal concentrations of disinfectants studied via a transcriptomic analysis using transcriptome sequencing (RNA‐*seq*). Applied and Environmental Microbiology, 83, AEM.01643–17. 10.1128/AEM.01643-17 PMC5717214 29030437

[mbo3720-bib-0038] Sousa, A. M. , & Pereira, M. O. (2014). *Pseudomonas aeruginosa* diversification during infection development in cystic fibrosis lungs: A review. Pathogens, 3, 680–703. 10.3390/pathogens3030680 25438018 PMC4243435

[mbo3720-bib-0039] Stryjewski, M. E. , & Corey, G. R. (2014). Methicillin‐resistant *Staphylococcus aureus*: An evolving pathogen. Clinical Infectious Diseases, 58(Suppl 1), S10–S19. 10.1093/cid/cit613 24343827

